# Milk-Alkali Syndrome: A Rare But Rising Cause of Hypercalcemia

**DOI:** 10.7759/cureus.88192

**Published:** 2025-07-17

**Authors:** Vikram Oke, Huda Khan, Moye Mathew, Praveen Datar, Karthik Iyer

**Affiliations:** 1 Pulmonary and Critical Care, Mercy Hospital Jefferson, Festus, USA; 2 Critical Care Medicine, Mercy Hospital Jefferson, Festus, USA; 3 Internal Medicine, Mercy Hospital Jefferson, Festus, USA

**Keywords:** acute kidney injury, hypercalcemia, metabolic alkalosis, milk-alkali syndrome, over-the-counter calcium-containing medications

## Abstract

Milk-alkali syndrome is a condition caused by ingesting an excessive amount of calcium and absorbable alkali. It is described by the triad of elevated blood calcium levels, acute kidney injury, and metabolic alkalosis. While it was historically rare, it is starting to become more common due to the use of over-the-counter calcium-containing medications for the treatment of acid reflux and osteoporosis. We present a case of a 51-year-old male who presented with altered mental status and generalized weakness. He was noted to have severe hypercalcemia and acute kidney injury with a history of recently increasing his calcium carbonate antacid for indigestion. He was appropriately treated with the resolution of hypercalcemia and presenting symptoms. Once considered rare, milk-alkali syndrome is now the third most common cause of hypercalcemia and should be included high in the differential diagnosis of hypercalcemia.

## Introduction

Milk-alkali syndrome is characterized by the classical triad of metabolic alkalosis, hypercalcemia, and acute kidney injury due to excessive intake of combined calcium and absorbable alkali [1].

In the majority of cases, milk-alkali syndrome is asymptomatic, diagnosed incidentally based on laboratory evidence of hypercalcemia, metabolic alkalosis, and renal failure. However, patients can present with symptoms suggestive of acute or chronic hypercalcemia depending on the severity and duration of exposure [2].

Neurological symptoms include headache, vertigo, malaise, and psychosis. Gastrointestinal symptoms include nausea, vomiting, and constipation. Genitourinary symptoms could include polyuria and nephrolithiasis [1].

The exact pathophysiology of milk-alkali syndrome is unknown; however, hypercalcemia is thought to play a major role. Renal insufficiency is a strong risk factor [3].

Milk-alkali syndrome was first identified in the 20th century and attributed to excessive use of milk with an absorbable alkali such as sodium bicarbonate, which, at that time, was the preferred treatment for peptic ulcer disease (PUD). After the adoption of proton pump inhibitors and histamine receptor antagonists for the treatment of PUD, cases of milk-alkali syndrome significantly decreased [1]. Recently, the number of reported cases seems to be rising, likely due to an increased use of over-the-counter calcium-containing medications for the treatment of acid reflux and osteoporosis.

## Case presentation

A 51-year-old male with a past medical history of hypertension, type 2 diabetes mellitus, gastro-esophageal reflux disease, and alcohol dependence, with a recent increase to half a bottle of vodka per day, presented with a two-month history of dizziness, visual disturbances, fatigue, confusion, and hallucinations worsening over three days. He started taking 15-20 calcium carbonate tablets daily for dyspepsia (each tablet containing approximately 200 mg of elemental calcium), translating to a daily calcium intake of 3,000-4,000 mg. He was also taking lisinopril-hydrochlorothiazide (20 mg-12.5 mg tablet) orally daily for management of hypertension and metformin 1,000 mg orally daily for diabetes.

On admission, the patient was alert, oriented, but slow to respond, hypertensive with blood pressure at 151/92 mmHg, while other vital signs were stable. Table 1 summarizes labs obtained by PCP two weeks prior to presentation to the hospital.

**Table 1:** Pertinent labs obtained by patients PCP, two weeks prior to presentation BUN: blood urea nitrogen; PCP: primary care physician; WBC: white blood cell

Lab Test	Reference Range	Value
WBC (k/µL)	4-11	7.8
Hemoglobin (g/dL)	13.5-17.0	16.9
Platelets (k/µL)	150-400	180
BUN (mg/dL)	7-25	18
Creatinine (mg/dL)	0.70-1.30	0.65
Calcium (mg/dL)	8.6-10.3	9.5
Bicarb (mmol/L)	20-32	28

Table 2 summarizes pertinent labs obtained at the time of presentation to the hospital.

**Table 2:** Pertinent labs obtained at the time of presentation to the hospital BUN: blood urea nitrogen; PTH: parathyroid hormone; PTHrP: parathyroid hormone-related protein; WBC: white blood cell

Lab Test	Reference Range	Value
WBC (k/µL)	4-11	12.3
Hemoglobin (g/dL)	13.5-17.0	18.5
Platelets (k/µL)	150-400	232
BUN (mg/dL)	7-25	31
Creatinine (mg/dL)	0.70-1.30	1.08
Calcium (mg/dL)	8.6-10.3	22.5
PTH (pg/mL)	15-65	10.2
PTHrP (pg/mL)	11-20	8
25-OH vitamin D (ng/mL)	30-100	11

**Figure 1:**
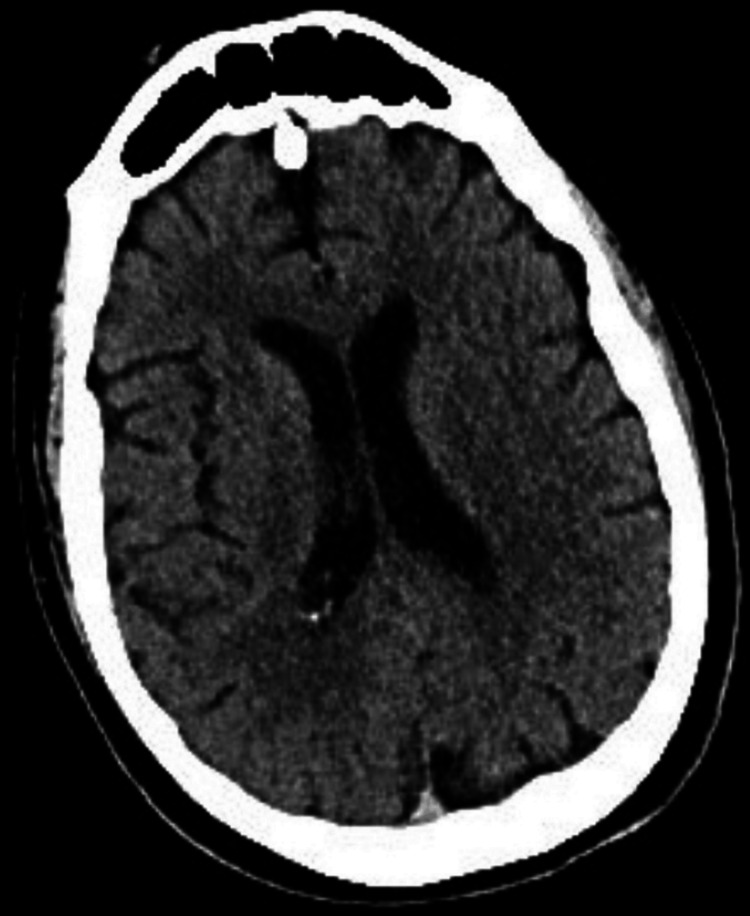
CT head obtained at admission revealed no acute intracranial process

**Figure 2:**
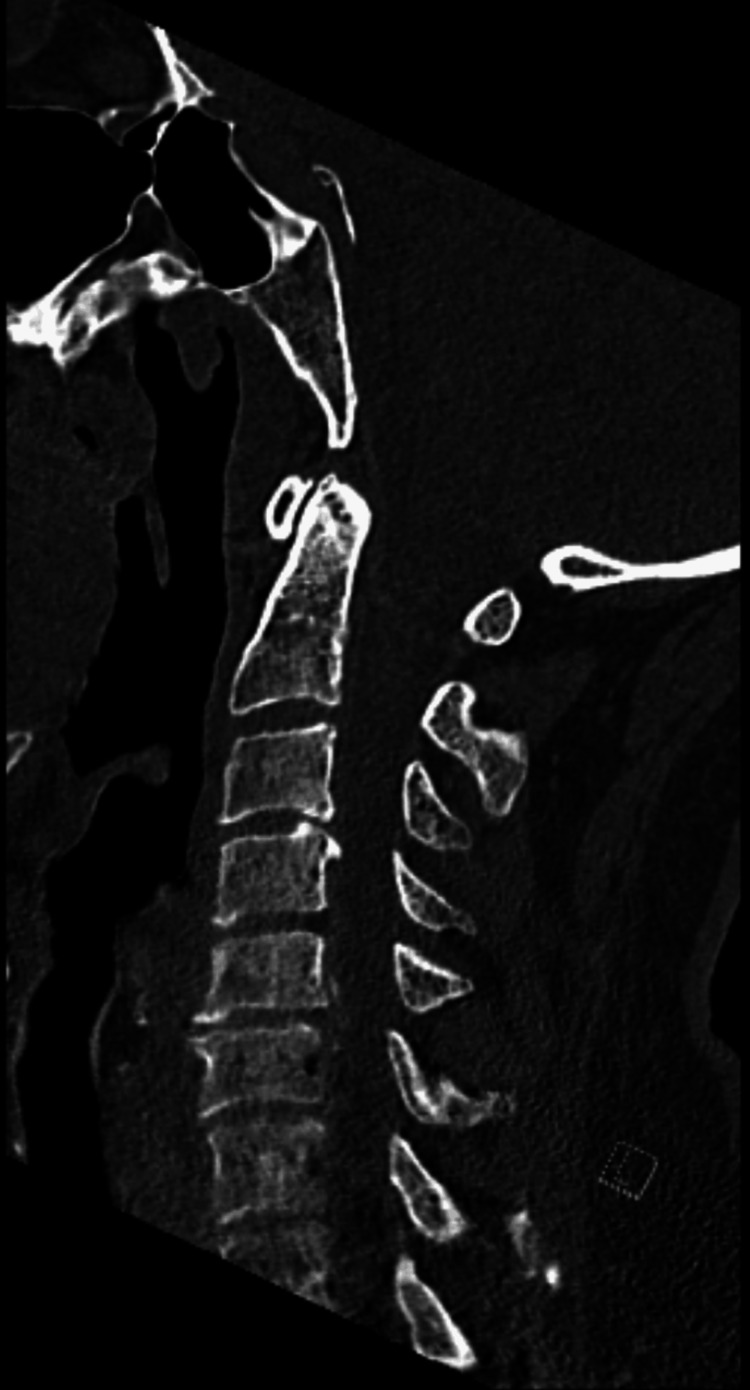
CT cervical spine obtained at admission with no evidence of cervical fracture or dislocation

**Figure 3:**
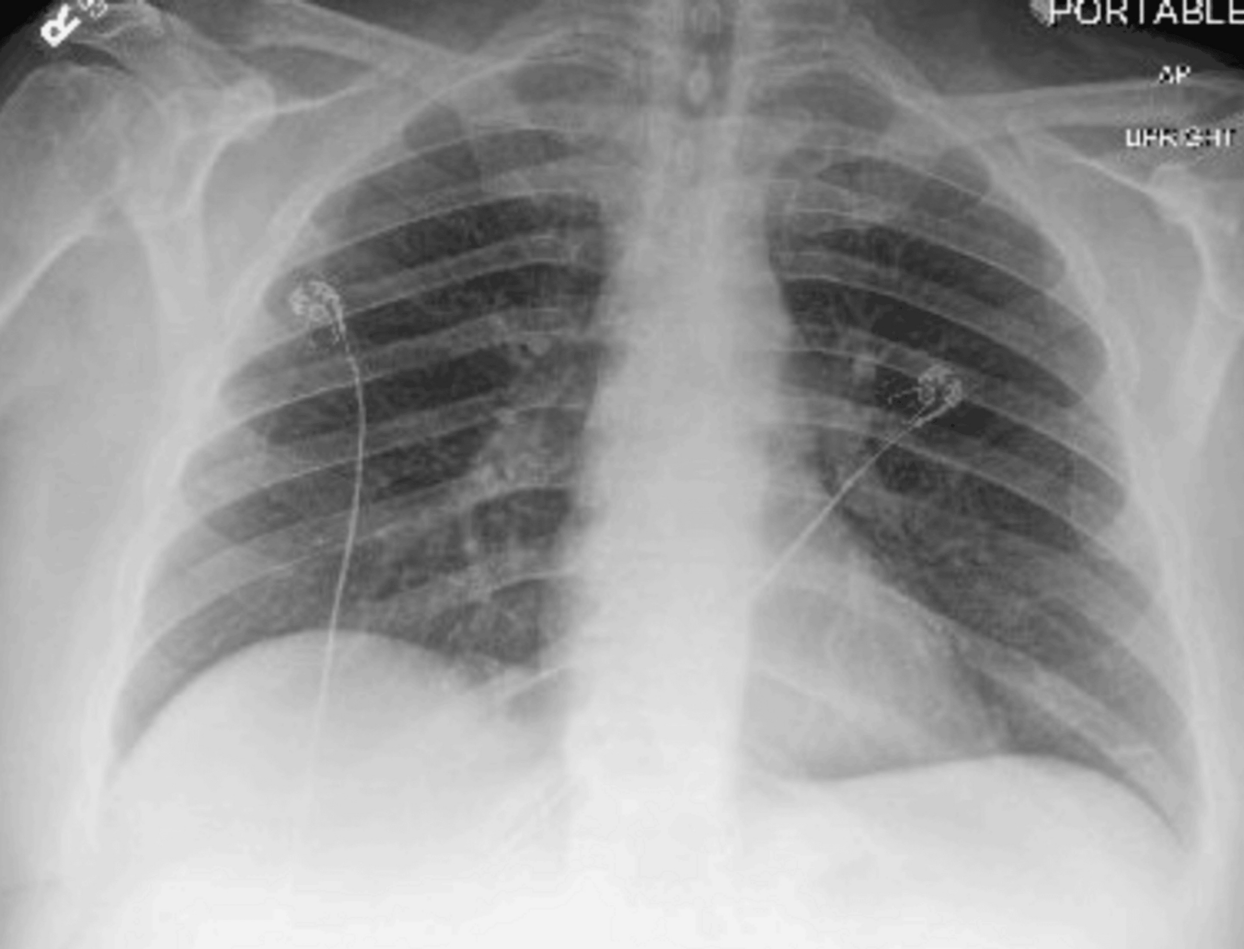
Chest X-ray at admission with no active pulmonary disease

**Figure 4:**
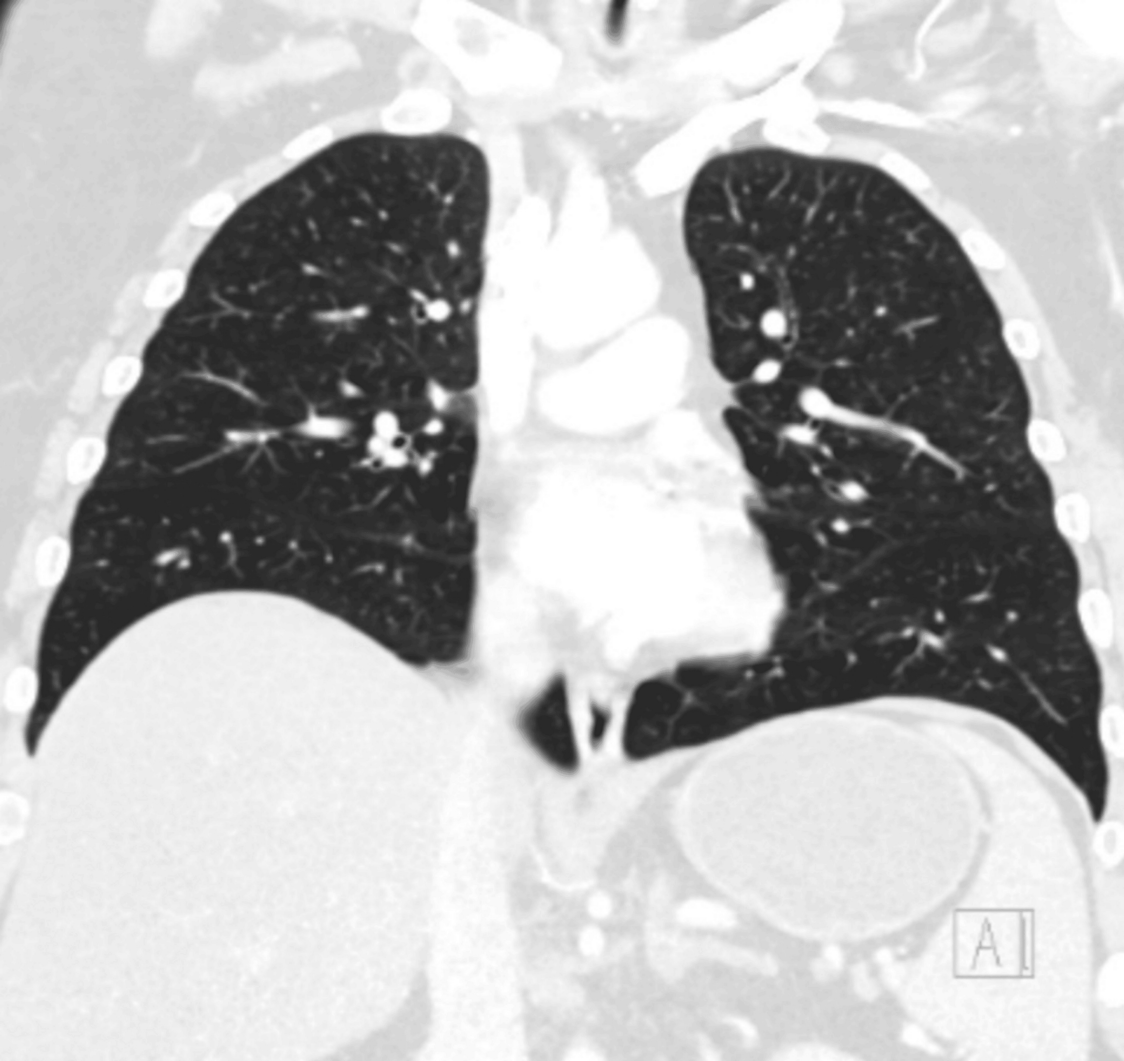
CT chest at admission with no pulmonary consolidation, nodule, mass, effusion, and no acute abnormality noted

**Figure 5:**
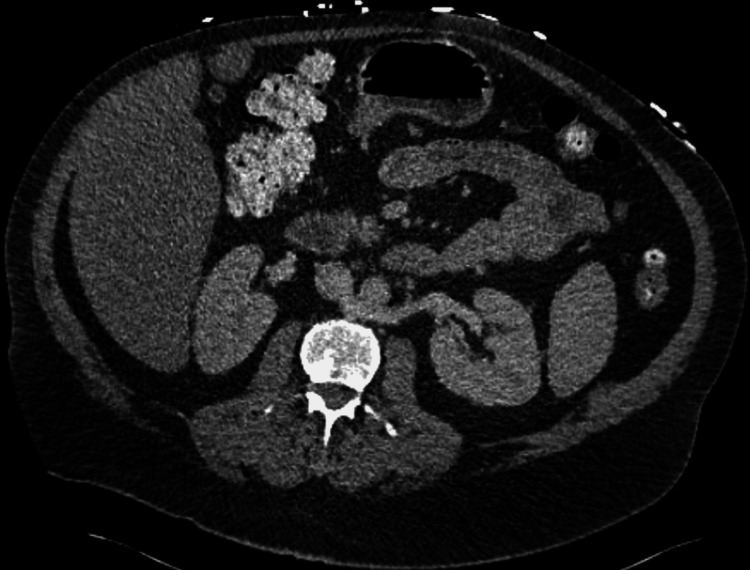
CT abdomen pelvis at admission with no acute abnormality

**Figure 6:**
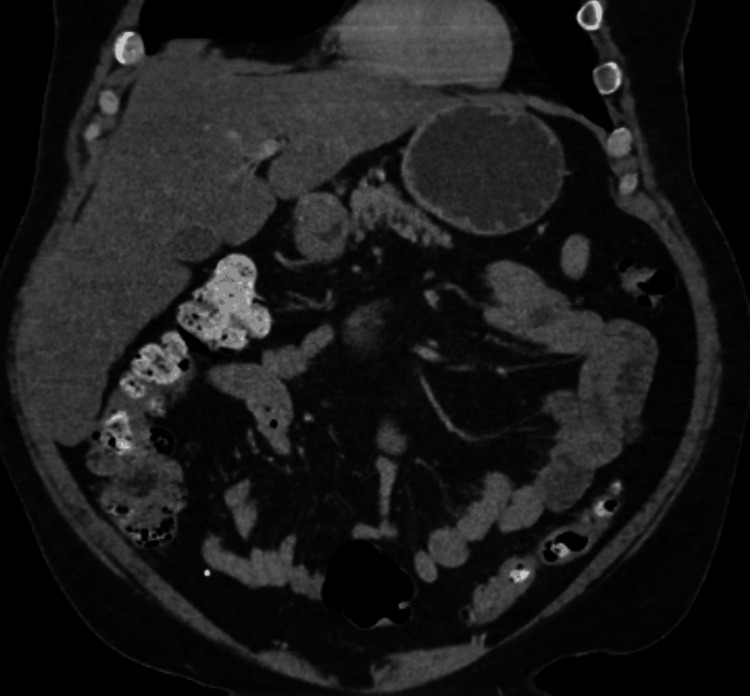
CT abdomen pelvis at admission (coronal view) with no acute abnormality

**Figure 7:**
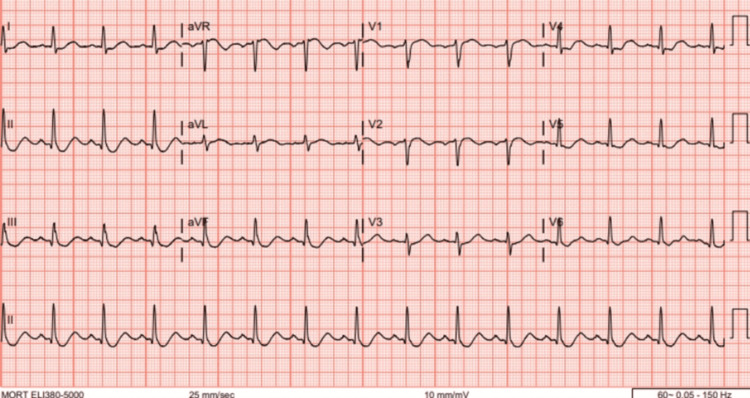
EKG at admission with sinus rhythm

Laboratory analysis showed severe hypercalcemia with total calcium level at 22.5 mg/dL, corrected for albumin, severe hyponatremia at 124 mmol/L, elevated bicarbonate of 39 mmol/L, and hypokalemia at 3.0 mmol/L. Creatinine was 1.08 mg/dL, elevated from the patient’s baseline of 0.65 mg/dL. Blood urea nitrogen (BUN) was elevated at 31 mg/dL, and the blood glucose level was 196 mg/dL. Parathyroid hormone (PTH) was low at 10.2 pg/mL. PTH-related protein (PTHrP) and 25-OH vitamin D levels were low. Complete blood count (CBC) revealed a white blood cell count of 12.3 k/μL and hemoglobin of 18.5 g/dL. The patient was started on isotonic intravenous fluids. CT chest, abdomen and pelvis with IV contrast, chest X-ray, and non-contrast head and cervical spine CT performed by an emergency room physician were unremarkable. The electrocardiogram did not reveal any changes suggestive of hypercalcemia (Figures 1-7). Two weeks prior to presentation, labs ordered by the primary care physician (PCP) were unremarkable. This included a serum calcium level, measured to be 9.5 mg/dL, corrected for albumin.

The patient was admitted to the ICU and treated with aggressive IV fluid resuscitation with isotonic fluids. Targeted hypercalcemia treatment included IV zoledronic acid (4 mg) and calcitonin. Home hydrochlorothiazide and antacids were discontinued. Renal function was closely monitored along with serial calcium levels. Presenting symptoms and hypercalcemia resolved with this treatment regimen, and the patient was discharged six days later. Table 3 summarizes his gradual improvement in renal function and electrolytes during hospital stay.

**Table 3:** Labs during the patient's hospital course

Lab Test	Reference Range	Day 1	Day 2	Day 3	Day 4	Day 5	Day 6
Calcium (mg/dL)	8.4-10.2	22.5	13.08	10	8	7.5	7.8
Sodium (mmol/L)	136-145	124	130	134	135	139	138
Potassium (mmol/L)	3.5-5.1	3.0	3.3	3.2	3.5	3.3	3.3
Bicarbonate (mmol/L)	22-29	39	32	28.4	26	26	23
Chloride (mmol/L)	98-107	74	89	94	96	99	102
Arterial pH	7.35-7.45	7.64	7.55	7.47			
Blood urea nitrogen (mg/dL)	6-20	31	21.4	17	14	12	13
Creatinine (mg/dL)	0.67-1.17	1.08	0.95	0.95	0.91	0.9	0.81
Phosphorus (mg/dL)	2.5-4.5	2.6	0.8	1.9	2.5	2.1	2.3
Magnesium (mg/dL)	1.6-2.6	1.6	1.1	1.0	1.1	1.4	1.7

## Discussion

In 1915, Dr. Bertram Sippy proposed that gastric acidity was the driving force behind chronic peptic ulcer disease [4]. He designed a treatment regimen involving daily consumption of calcium-rich foods (e.g., milk and cream) and alkaline powders (e.g., sodium bicarbonate, bismuth carbonate, or magnesium oxide) to protect the gastric ulcer until it healed [4]. His treatment regimen was greatly successful, making “milk-alkali therapy” the landmark treatment for peptic ulcer disease. However, Mayo Clinic reported toxic effects of the treatment in patients requiring higher doses of alkali, including irritability, weakness, and headache [5]*.* In 1936, Cope linked this treatment to hypercalcemia, metabolic alkalosis, and renal insufficiency, thus establishing milk-alkali syndrome [6]. With the advent of proton pump inhibitors and H2 receptor blockers for the management of peptic ulcer disease, the syndrome became rare [1]. However, the recent rise in reported cases is attributed to higher use of calcium carbonate for acid reflux and osteoporosis. Calcium carbonate is also frequently prescribed to cardiac transplant recipients to prevent peptic ulcer disease and osteoporosis associated with immunosuppressive therapy [7]. Our patient excessively used over-the-counter calcium carbonate supplements for acid reflux.

Milk-alkali syndrome accounts for more than 10% of hypercalcemia and is now considered to be the third most common cause in hospitalized patients, after primary hyperparathyroidism and malignancy [2].However, it is a diagnosis of exclusion, and all causes of hypercalcemia should be considered first. Diagnosis requires a history of excessive calcium and alkali consumption, hypercalcemia, and acute kidney injury without alternate explanations. Most patients are asymptomatic, with hypercalcemia, metabolic alkalosis, and acute kidney injury typically discovered as incidental findings. Some patients, however, will present with acute or chronic signs of hypercalcemia [2]. The presentation occurs in three progressive stages - acute, subacute, and chronic - depending on the duration of calcium product consumption [1].

Acute hypercalcemia symptoms, which include nausea, vomiting, vertigo, and headache, present within a month. The subacute phase includes both acute and chronic symptoms. Chronic phase, typically seen in patients taking products such as antacids for years, may involve nephrocalcinosis, tremors, psychosis, and calcium deposition in organs and tissues [1]. Metastatic calcification to the lungs and liver has also been reported in long-term antacid users [8]. Our patient presented with a two-month history of dizziness, visual disturbances, fatigue, confusion, and hallucinations worsening over three days prior to presentation*. *His last alcohol drink was 15 hours prior to presentation to the hospital. He was monitored closely for alcohol withdrawal and administered lorazepam as needed, approximately 36 hours after admission for alcohol withdrawal symptoms.

While the exact pathophysiology of milk-alkali syndrome is unknown, elevated blood calcium level is thought to play a major role. Additionally, renal insufficiency is a strong risk factor [3]. It has also been suggested that large amounts of calcium ingestion can cause inadequate suppression of calcitriol in some individuals, leading to intestinal calcium hyperabsorption [9]. Increased blood calcium levels cause vasoconstriction and decrease glomerular filtration rate by indirectly inhibiting Na-K-2Cl channels in the medullary part of the thick ascending limb. It also causes a decrease in water reabsorption by inhibiting antidiuretic hormone receptors in the basolateral membrane of collecting tubules. The resultant hypovolemia and decreased GFR cause an increased renal bicarbonate reabsorption, leading to the development of metabolic alkalosis. Alkalosis itself contributes to hypercalcemia by increasing renal calcium reabsorption. Therefore, vicious self-reinforcing cycle develops from the elevated calcium, causing the kidney injury, which leads to alkalosis and a subsequent increase in calcium reabsorption [9]. Hypercalcemia can worsen in patients with renal insufficiency, given its role in causing acute kidney injury [3]. Our patient had acute kidney injury, an elevated bicarbonate level consistent with hypochloremic hypokalemic metabolic alkalosis, and hypercalcemia (refer to Table 3). Additionally, our patient was on an angiotensin-converting enzyme inhibitor and hydrochlorothiazide, which are risk factors in the development of acute kidney injury.

The main treatment of milk-alkali syndrome is typically supportive, including discontinuing the offending agent, aggressive IV fluid hydration, furosemide to enhance calcium excretion, and hemodialysis in refractory cases [10]. Hypercalcemia was successfully treated in our patient with IV fluids, calcitonin, zoledronic acid, discontinuation of calcium carbonate antacid, and thiazide. Our patient also presented with hyponatremia, which was secondary to volume depletion and corrected appropriately with IV hydration.

## Conclusions

Milk-alkali syndrome was once an adverse effect of PUD treatment, but now it is often due to an excessive consumption of calcium-containing antacids for dyspepsia and calcium supplements for osteoporosis. The exact mechanism of this syndrome is unknown, but the adverse effect on the kidneys suggests that hypercalcemia plays a major role. Prolonged hypercalcemia without treatment could potentially lead to permanent kidney damage and other complications. Treatment is supportive with aggressive hydration and removal of the offending agent. Once considered rare, milk-alkali syndrome is now the third most common cause of hypercalcemia and should be included in the differential diagnosis of hypercalcemia.
